# ORIHIME study: real-world treatment patterns and clinical outcomes of 338 patients with acquired hemophilia A from a Japanese administrative database

**DOI:** 10.1007/s12185-022-03467-w

**Published:** 2022-11-04

**Authors:** Yoshiyuki Ogawa, Kagehiro Amano, Yukari Matsuo-Tezuka, Norihiro Okada, Yoichi Murakami, Takao Nakamura, Haruko Yamaguchi-Suita, Keiji Nogami

**Affiliations:** 1 Department of Hematology, Gunma University Graduate School of Medicine, 3-39-22 Showa-machi, Maebashi, Gunma 371-8511 Japan; 2 Department of Laboratory Medicine, Tokyo Medical University, Tokyo, Japan; 3 Chugai Pharmaceutical Co., Ltd, Tokyo, Japan; 4 Department of Pediatrics, Nara Medical University, Kashihara, Japan

**Keywords:** Activities of daily living, Administrative claims, Hemophilia A, Japan, Rehabilitation

## Abstract

**Background:**

Acquired hemophilia A (AHA) is a rare disorder, and clinical practices for treating AHA have not been fully clarified in Japan.

**Objectives:**

This study aims to investigate the epidemiology of AHA and real-world treatment practices in Japan.

**Patients/methods:**

This observational study was based on a health administrative database of hospitalized patients diagnosed with AHA who were treated with immunosuppressants.

**Results:**

The study included 214 males and 124 females (mean age 75.7 years). The most frequently used bypassing agent was recombinant activated factor VII. The predominant choice of immunosuppressant for first-line treatment was steroid monotherapy. Median days from the index date to the start of rehabilitation was 65.0 for cardiovascular, 35.5 for respiratory and 23.0 for locomotor. The proportion of patients with an activities of daily living (ADL) score < 70 points was high at both first admission and final discharge (47.4% and 38.8%). The percentage of deaths during hospitalization was 18.6%.

**Conclusions:**

This study clarified the treatment patterns and clinical outcomes of AHA in a large population in Japan. This was the first study showing ADL score distribution and time to rehabilitation. Further investigation is needed to develop better clinical practices for treatment of AHA.

**Supplementary Information:**

The online version contains supplementary material available at 10.1007/s12185-022-03467-w.

## Background

### Pathology

Acquired hemophilia A (AHA) is a bleeding disorder characterized by the formation of autoantibodies against endogenous coagulation factor VIII (FVIII) in an acquired manner, resulting in a marked decrease of FVIII activity and presenting with bleeding episodes such as spontaneous subcutaneous hemorrhage, intramuscular hemorrhage and occasional occurrence of serious hemorrhages. The prognosis of AHA is poor, and mortality of early onset AHA is high. The cause of death is usually serious hemorrhage or severe infection caused by the immunosuppression to eliminate autoantibodies [1]. Although approximately more than a half of AHA cases are idiopathic without identifiable associated conditions, AHA is known to be associated with malignancy, autoimmune diseases, dermatological diseases, pregnancy, delivery, etc. [2, 3].

### Epidemiology

In light of some large surveys of AHA populations to date, including the European Acquired Haemophilia Registry (EACH2) [4], GTH-AH 01/2010 study [5] and the Surveillance des Auto antiCorps au cours de l’Hémophilie Acquise (SACHA) registry [6] alongside some epidemiological results and previous surveillances, it showed that the estimated incidence of AHA was 1.34 to 1.48 per million people per year in the United Kingdom [7, 8]. In previous surveys, the male to female ratios were 1:0.7 to 1.3 [4, 5, 8]. The onset ages ranged widely from 12 to 85 years (median 70 years), of which nearly 90% of patients were over 50 years old [1, 9], and childhood onset was uncommon [10, 11]. Unlike in Europe, epidemiological studies of AHA in Japan have been limited. Only two have been conducted in the past couples of decades, including a 3-year cohort-study of 55 patients in 2008 [1] and a single-center observational study of 25 patients [12].

### IST and hemostatic treatment

The treatment of AHA requires immunosuppressive therapy (IST) to eliminate autoantibodies and hemostatic treatment for bleeding. The first line treatment of IST for AHA is prednisolone (PSL) monotherapy or a combination of PSL and cyclophosphamide (CPA) in the Japanese guideline [13]. Selection of initial dose of immunosuppressive agents and the timing of dose reduction should be carefully considered for each individual patient to manage the risk of infectious diseases or exacerbating underlying diseases such as diabetes [13]. In addition to IST, hemostatic treatment is also required for bleeding symptoms [13]. Bypassing agents are widely used for the hemostatic treatment of AHA and comprised of recombinant activated factor VII (rFVIIa), activated prothrombin complex concentrate (aPCC) [1] and plasma-derived factor VIIa and factor X (FVIIa/FX) launched only in Japan [13]. Although these bypassing agents exert supposedly sufficient hemostatic effects, they are not always successful in managing bleeding symptoms [1, 2, 7, 9, 14, 15]. Understanding the incidence of thromboembolic events in AHA patients is important [13, 16] because AHA patients often have multiple thrombotic risk factors, including advanced age, concurrent diseases such as malignancies and autoimmune diseases, hemostatic medication use, an overshoot of FVIII activity after complete remission and prolonged bed rest.

### Rehabilitation and ADL

Many AHA patients are the elderly and often require bed rest during bleeding episodes when serious organ bleeding and intramuscular/intra-articular (especially intramuscular) bleeding occur [15]. Since long-term bed rest can lead to a decrease in activities of daily living (ADL), an early start of rehabilitation is recommended after hemorrhage is controlled. Recent studies reported that some AHA patients who were refractory to immune suppressive therapy benefited from the use of prophylactic APCC [17], and the emerging literature on the use of prophylactic emicizumab [18] started to change clinical practices in acquired hemophilia. On the other hand, as previously mentioned, hemostatic management with bypassing agents is not always successful, and prophylactic use of bypassing agents to AHA patients during rehabilitation period is not recommended because of the lack of evidence for its efficacy [13]. Therefore, the optimization of rehabilitation and ADL-sustaining are urgent issues on AHA.

### Objective and significance of this study

The objective of this study was to investigate the epidemiology and treatment patterns of AHA in clinical practices in Japan. The difficulty in collecting cases of rare disease was solved by using a large administrative database containing information from multiple medical institutions. This is the first large-scale study to investigate the epidemiology and treatment patterns of AHA patients in Japan.

## Methods

### Database

We used a health administrative database of hospitals subject to the Diagnosis Procedure Combination (DPC)/Per-Diem Payment System (PDPS), which is a variation of the Diagnosis-Related Group/Per Diem Payment System developed earlier in the United States. Basically, per diem payment is used for hospitalization in DPC hospitals, whereas other medical costs are calculated based on the fee-for-service basis [19, 20]. The database used in this study is owned by Medical Data Vision Co., Ltd (MDV; Tokyo, Japan). It is one of the largest administrative database for commercial use in Japan and contains abundant treatment information data. The database covers 31.8 million patients (as of the end of May 2020) [21] from 413 facilities (approximately 24% of acute-care hospitals in Japan), which use the DPC/PDPS system. In addition, the objective to collect data on patients requiring in-hospital care fits the characteristics of MDV data, which covers a relatively large proportions of acute-care hospitals.

Personal patient information within the MDV database was protected because it was anonymized after the secondary use permission from the provider hospitals was obtained.

### Ethical matters

The study protocol was reviewed and approved by the Ethical Committee (MINS Institutional Review Board). The study was conducted in compliance with the Japanese Ethical Guidelines for Medical and Health Research Involving Human Subjects. And this study was registered on University Hospital Medical Information Network Clinical Trial Registry (UMIN000043479).

### Study design

This was a retrospective, observational study using an administrative data. The data period was from April 1, 2008 to March 31, 2020. The index date was defined as the date of the first hospitalization when an AHA (suspected cases were excluded) disease code was recorded in the receipt data. Receipt disease codes are unified standardized codes in Japan, and receipt disease code “8845658” has been designated as AHA prior to the AHA specific code defined in ICD-10. The end of the observation period was defined as the most recent month of treatment recorded for the patient, or the last day of the data period, whichever came first. The follow-up period was defined as the period from the index date to the end of the observation period. The baseline period was defined as 1 month before and after the index date. The start date of the baseline period was the start date of the observation period.

Patients who met all of the following 3 criteria were included: (1) patients with a disease diagnosis code for acquired coagulation factor deficiency (ICD-10 code: D684) and with a diagnosis of AHA (defined using the receipt disease code 8845658) (suspected cases were excluded); (2) patients who were admitted to the hospitals in the month of AHA diagnosis, and (3) patients who had immunosuppressive agents on or after the date of the first admission.

Patients with diagnosis of antiphospholipid syndrome, lupus anticoagulant, or some coagulation factor deficiencies for AHA definitive diagnosis were excluded (suspected cases were excluded for all diseases). All exclusion criteria were applied for the entire observation period. Patients who were diagnosed with antiphospholipid syndrome or lupus anticoagulant were excluded because the disease status was different from that of AHA [22, 23].

Concurrent diseases (suspected cases were excluded) were determined by medical experts after searching receipt disease names from the code list of the International Statistical Classification of Diseases and Related Health Problems 10th Revision (ICD-10) (2013). Each medical product name was identified by the generic name. Medical activity was determined by medical experts after searching from receipt names of medical care activity.

### Outcomes

Outcomes included treatment/implementation status (hemostatic therapy, IST, transfusion, plasmapheresis therapy, and rehabilitation) and the clinical outcomes such as the frequency of hospital admissions, death, bleeding, thromboembolism, infections, and ADL [Barthel Index (BI)] [24] in AHA patients during the hospitalization period.

Rehabilitations were defined based on the insurance claims code during hospitalization, and the descriptions of rehabilitations were listed as follows: (1) disuse syndrome: rehabilitation to improve dysfunctions such as joint contractures, cardiopulmonary dysfunction, digestive dysfunction, intellectual inactivity, etc., which occur secondary to physical inactivity; (2) locomotor: rehabilitation to improve joint disorders caused by rheumatoid arthritis, knee osteoarthritis, etc., and spinal disorders, back pain, stiff shoulders and locomotor disorders caused by sports, etc.; (3) cerebrovascular: rehabilitation for the purpose of recovery of basic movement ability impaired by cerebrovascular diseases, central nervous system diseases, etc.; (4) cardiovascular: rehabilitation to recover cardiac function and prevent recurrence of diseases; (5) respiratory: rehabilitation to recover or maintain respiratory function in patients with disorders due to respiratory diseases.

### Statistical analysis

Data collection was managed according to MDV’s standard procedures. All statistical analyses were performed using SAS® ver. 9.4 (SAS Institute Inc., Cary, NC, USA).

Unless otherwise noted, continuous variables were summarized by using the number of patients, arithmetic mean, standard deviation (SD), median, interquartile range (IQR), minimum, and maximum values as descriptive statistics. The follow-up period was covered from the index date to the end of the observation period.

Prednisolone equivalents were used for steroids dosing amounts. Weight on the index date was used, and patients with missing weight values were excluded from all weight-related analyses.

For patients who were admitted to hospital multiple times during the follow-up period, the length of each hospitalization was added up.

In the analysis of ADL scores, patients with even one missing item of ADL scores were excluded from the calculation, and missing data was not imputed. In the same score analysis, the hospitalization data on the index date was used for the first admission, and the latest discharge data after the index date was used for the final discharge if multiple hospitalizations were recorded. The incidence/100 person-year analyses and admission/discharge related analyses included 328 patients, excluding 10 patients with missing data. Only rehabilitations covered by the medical insurance were captured in this medical administrative database, and those categorizations (cerebrovascular and disuse syndrome etc.) were dependent on types of receipts. The incidence rate of hospital admission/discharge, bleeding, infection, and hemostasis for each patient was calculated based on the total follow-up period (person-year). On the status of hospitalization/discharge, summary data on discharge was used, and in-patients on data cut-off were not included in the analysis.

### Results

### Patient demographics (Fig. 1 and Table 1)

**Fig. 1:**
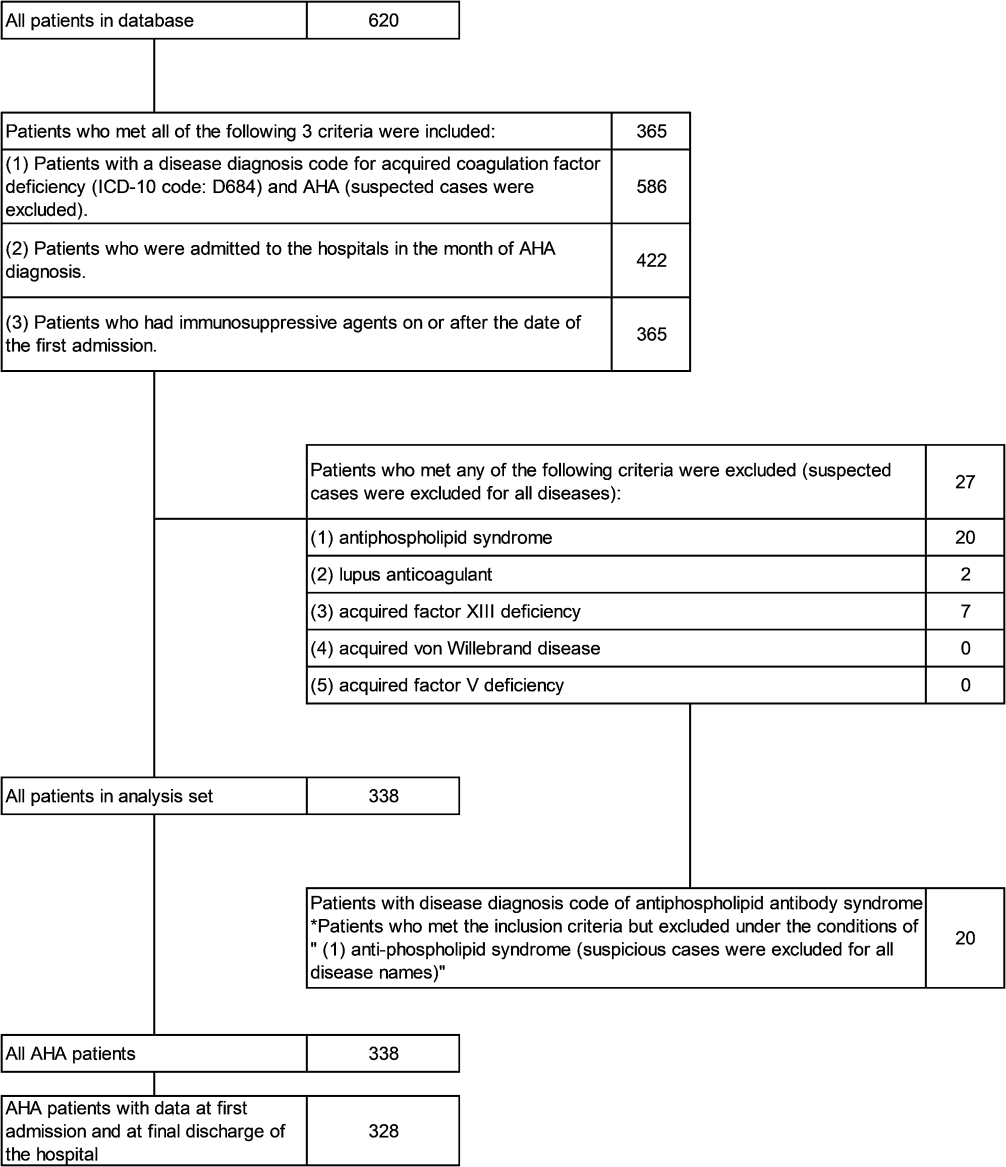
shows the disposition of all patients included in the MDV database. A total of 620 patients were included in the database, and 338 patients were included in the AHA patient population after applying inclusion and exclusion criteria

**Table 1:** Baseline characteristics

Analysis set: All patients						
	Overall		Male		Female	
	*N* = 338		*N* = 214		*N* = 124	
	*n*	(%)	*n*	(%)	*n*	(%)
Age						
Mean (SD)	75.7(12.3)		74.2(10.8)		78.3(14.1)	
Min	21		32		21	
Median	78.0		75.0		82.0	
Max	96		95		96	
0–40	7	(2.1)	2	(0.9)	5	(4.0)
41–50	8	(2.4)	6	(2.8)	2	(1.6)
51–60	17	(5.0)	13	(6.1)	4	(3.2)
61–70	71	(21.0)	57	(26.6)	14	(11.3)
71–80	87	(25.7)	62	(29.0)	25	(20.2)
81–90	129	(38.2)	69	(32.2)	60	(48.4)
91–100	19	(5.6)	5	(2.3)	14	(11.3)
Hospital department						
Internal medicine	167	(49.4)	95	(44.4)	72	(58.1)
Hematology	76	(22.5)	56	(26.2)	20	(16.1)
Hematology oncology	15	(4.4)	6	(2.8)	9	(7.3)
Gastroenterological internal medicine	9	(2.7)	8	(3.7)	1	(0.8)
Orthopedics	8	(2.4)	3	(1.4)	5	(4.0)
General surgery	7	(2.1)	6	(2.8)	1	(0.8)
Others	56	(16.6)	40	(18.7)	16	(12.9)
		*N* = 328^a^				
The sum of follow-up times for each patient [person-year]		507.02				

^a^Of the analysis population (*N* = 338), 10 patients had no summary data at hospital discharge

The patients number of this section is *N* = 328

A total of 620 patients diagnosed with acquired coagulation factor deficiency were included in the database covering a period from April 2008 to March 2020. Of these, 338 patients were included in the study population after inclusion and exclusion criteria were applied. The patient flow chart is shown in Fig. 1.

The cohort of 338 patients at baseline comprised 214 males and 124 females with an overall mean age (S.D.) of 75.7 years (12.3) (median 78.0) ranging from 21 to 96 years. The most common age group was 81–90 years, accounting for 38.2% (129 patients). As for hospital departments, percentage of AHA patients visiting on the index date was recorded as follows: 49.4% of internal medicine (167 patients), 22.5% of hematology (76 patients) and 4.4% of hematology oncology (15 patients) (Table 1).

The median duration (Q1/Q3) of cumulative hospitalization period was 62.0 (35.0/101.5) days (328 patients). The observation period was from the start of the baseline period to the end of the most recent month of recorded treatment or the last day of the data period. Median (Q1/Q3) (mean) of the observation period for all patients (*N* = 338) was 276.5 (76.0/820.0) days (mean [SD] 550.0 days [628.4]).

### Concurrent diseases (Table 2)

**Table 2:** Concurrent diseases, pregnancy/delivery for all patients and those of in-hospital death cases

Analysis set: All patients		
Classification	*N* = 338	Classification
Disease name	*n*	(%)
Possible idiopathic	148	(43.8)
Concurrent diseases	190	(56.2)
Pregnancy/delivery^a^	0	(0.0)
Malignancy	61	(18.0)
Malignant lymphoma	13	(3.8)
Colon cancer	11	(3.3)
Prostate cancer	10	(3.0)
Stomach cancer	8	(2.4)
Autoimmune disease	40	(11.8)
Rheumatoid arthritis	13	(3.8)
Dermatological disease	18	(5.3)
Pemphigus	11	(3.3)
Pemphigoid	11	(3.3)
Other	134	(39.6)
Diabetes mellitus	114	(33.7)
Hepatitis	22	(6.5)
Bronchial asthma	14	(4.1)

Disease names were sorted by each classification in descending order of the patient numbers and only the most frequent diseases were shown

The most frequently recorded categories of concurrent diseases were shown, and the disease names recorded in more than 2% of all patients were shown in this table

^a^Percentage of total female patients (*N* = 124)

Approximately more than a half of the patients (56.2%, 190 patients) had some concurrent diseases defined in the month the index date was registered in the database. The remaining patients (43.8%, 148 patients) were possible idiopathic cases. The percentages of patients with concurrent diseases by disease classifications were 18.0% (61 patients) for malignancy, 11.8% (40 patients) for autoimmune diseases, 5.3% (18 patients) for dermatological diseases, and 39.6% (134 patients) for others. Among malignancies, malignant lymphoma was the most frequently reported (3.8%, 13 patients), followed by colon cancer (3.3%, 11 patients) and prostate cancer (3.0%, 10 patients). Among autoimmune diseases, rheumatoid arthritis (RA) was the most frequently reported (3.8%, 13 patients). Among dermatological diseases, pemphigus and pemphigoid were the most frequently reported (3.3%, 11 patients). Among others, diabetes mellitus was the most frequently reported (33.7%, 114 patients). None of the patients had events related to pregnancy or delivery (Table 2).

### Hemostatic agent (Table 3)

**Table 3:** Use of hemostatic agents

Analysis set: All patients										
Type of hemostatic agent	*N* = 338		Total initial dose on the first date				Duration of administration			
			(µg/kg, U/kg or mg/kg)				(Day)			
	*n*	(%)	*n*	Q1	Median	Q3	*n*	Q1	Median	Q3
Bypassing agent	153	(45.3)	–	–	–	–	–	–	–	–
rFVIIa (μg/kg)	129	(38.2)	118	121.36	221.98	319.15	129	2.0	5.0	10.0
aPCC (U/kg)	36	(10.7)	34	68.18	91.59	150.00	36	4.0	6.0	8.5
FVIIa/FX (μg/kg)	14	(4.1)	13	87.80	104.00	109.86	14	1.0	2.5	4.0
FVIII agent	8	(2.4)	–	–	–	–	–	–	–	–
FVIII agent (U/kg)	8	(2.4)	8	17.43	27.99	35.54	8	1.0	2.5	5.0
Tranexamic acid	116	(34.3)	–	–	–	–	–	–	–	–
Tranexamic acid (mg/kg)	116	(34.3)	110	13.33	18.81	26.53	116	5.5	13.0	35.0
DDAVP	2	(0.6)	–	–	–	–	–	–	–	–
Desmopressin acetate hydrate (μg/kg)	2	(0.6)	2	0.25	0.35	0.46	2	1.0	1.5	2.0

If weight or dosage data are missing, the initial dosing amount for the patient was not calculated

Days of the drug administration during hospitalization were counted and if once the administration was interrupted then re-started, days of the interrupted periods were not included in the calculation, and only days of drug administration were added

*FVIII* factor VIII, *rFVIIa* recombinant activated factor VII, *aPCC* activated prothrombin complex concentrate, *FVIIa/FX* plasma-derived factor VIIa and factor X, *DDAVP* desmopressin acetate hydrate

Among the 4 hemostatic agent groups (bypassing agents, FVIII agents, tranexamic acids, and DDAVP), bypassing agents were the most frequently used with 45.3% (153 patients) followed by tranexamic acid with 34.3% (116 patients). FVIII agents and DDAVP were relatively less used (8 and 2 patients, respectively). Focusing on the analysis of bypassing agent use, rFVIIa was the most frequent with 38.2% (129 patients), followed by aPCC with 10.7% (36 patients) and FVIIa/FX with 4.1% (14 patients). The median total initial dose (Q1/Q3) on the first date was 221.98 (121.36/319.15) μg/kg for rFVIIa, 91.59 (68.18/150.00) U/kg for aPCC, and 104.00 (87.80/109.86) μg/kg for FVIIa/FX. The median duration of administration was 5.0 (2.0/10.0) days for of rFVIIa, 6.0 (4.0/8.5) days for of aPCC and 2.5 (1.0/4.0) days for FVIIa/FX (Table 3). The number of doses was 2.0 times (per day) in maximum for all hemostatic agents. Since some patients had the records of multiple hemostatic agents use in the same periods, the overall usage rate exceeded 100%.

Transfusions were conducted with human RBC fluid (53.8%, 182 patients) and fresh frozen human plasma (20.7%, 70 patients).

Plasmapheresis therapy was rare (1.5%, 5 patients).

### IST (Table 4)

**Table 4:** Use of immunosuppressive agents

Analysis set: all patients										
Immunosuppressive treatment line	*N* = 338		Total initial dose on the first date				Duration of administration			
			(µg/kg, U/kg or mg/kg)				(Day)			
	*n*	(%)	*n*	Q1	Median	Q3	*n*	Q1	Median	Q3
Overall	338	(100.0)	–	–	–	–	338	59.0	158.0	455.0
1st line										
(1) Steroid monotherapy	292	(86.4)	–	–	–	–	292	23.0	82.5	246.5
PSL (injection)	119	(35.2)	72	0.64	0.89	0.99	119	5.0	10.0	24.0
mPSL (injection)	38	(11.2)	18	1.88	12.50	15.55	38	3.0	3.0	6.0
PSL (oral)	258	(76.3)	183	0.43	0.86	0.98	258	28.0	98.0	273.0
mPSL (oral)	1	(0.3)	1	0.16	0.16	0.16	1	7.0	7.0	7.0
(2) PSL + CPA (oral)	30	(8.9)	–	–	–	–	30	57.0	173.5	550.0
(3) PSL + CPA (injection)	3	(0.9)	–	–	–	–	3	51.0	104.0	237.0
(4) PSL + CyA	4	(1.2)	–	–	–	–	4	388.5	756.0	907.5
(5) CPA monotherapy	2	(0.6)	–	–	–	–	2	10.0	12.0	14.0
(6) RTX monotherapy	1	(0.3)	–	–	–	–	1	6.0	6.0	6.0
(7) RTX combination	3	(0.9)	–	–	–	–	3	71.0	78.0	86.0
(8) Others	3	(0.9)	–	–	–	–	3	7.0	87.0	314.0

The use of multiple IST was recorded in one patient, so the overall usage rate exceeded 100%

If weight or dosage data are missing, the initial dosing amount for the patient was not calculated

–: Data are not applicable for the combination name

*mPSL* methyl-prednisolone, *PSL* prednisolone, *CPA* cyclophosphamide, *CyA* cyclosporine A, *RTX* rituximab

Regarding product analysis among the 8 IST drug groups (i.e., steroid monotherapy, PSL + CPA oral, PSL + CPA injection, PSL + cyclosporin A [CyA], CPA monotherapy, rituximab [RTX] monotherapy, RTX combination, and others), the predominant choice of immunosuppressant for first-line treatment was steroid monotherapy (86.4%, 292 patients). The steroid monotherapy group included PSL (oral) with 76.3% (258 patients). The median durations of oral drug administrations were 98.0 days for PSL and 173.5 days for PSL + CPA (Table 4). Since the use of multiple IST was recorded in one patient, the overall usage rate exceeded 100%.

### Rehabilitation (Table 5)

**Table 5:** Implementation status of rehabilitation

Analysis set: All patients					
Category of rehabilitation	*N* = 338		From index date Time to initiation (day)		
	*n*	(%)	Q1	Median	Q3
Rehabilitation	223	(66.0)	–	–	–
Disuse syndrome	104	(30.8)	6.0	16.5	37.5
Locomotor	73	(21.6)	7.0	23.0	59.0
Cerebrovascular	49	(14.5)	6.0	19.0	80.0
Respiratory	24	(7.1)	13.5	35.5	123.5
Cardiovascular	6	(1.8)	6.0	65.0	119.0
No rehabilitation	115	(34.0)	–	–	–

Rehabilitation was most commonly implemented for disuse syndrome in 104 patients (30.8%), followed by locomotor rehabilitation in 73 patients (21.6%), cerebrovascular in 49 patients (14.5%), respiratory in 24 patients (7.1%), and cardiovascular in 6 patients (1.8%). Median days (Q1/Q3) from the index date to the start of rehabilitation was 16.5 (6.0/37.5) for disuse syndrome, 23.0 (7.0/59.0) for locomotor, 19.0 (6.0/80.0) for cerebrovascular, 35.5 (13.5/123.5) for respiratory, and 65.0 (6.0/119.0) for cardiovascular (Table 5).

### ADL (Fig. 2 and Table S1)

**Fig. 2:**
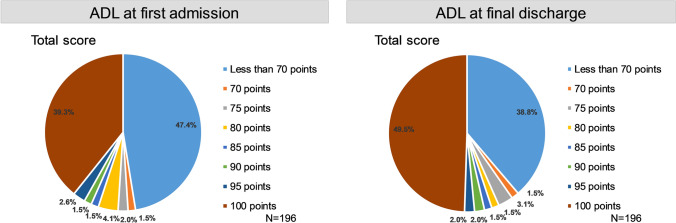
shows the composition of ADL scores (Barthel Index) at first admission and final discharge for all evaluable patients (*N* = 196) with no missing data for all 10 items

ADL scores from BI [24] on first hospitalization and final discharge were compared. In the 196 patients with data for all 10 ADL scores, the proportion of full scores increased more at final discharge for all items compared with the first admission. Scores between 70 and 100 points were considered as independent, and under 70 points were deemed as non-independent [25]. The proportions of patients with a total ADL score of less than 70 points was 47.4% (93 patients) at first admission and 38.8% (76 patients) at final discharge (Fig. 2 and Table S1).

### Mortality (Table 6)

**Table 6:** Concurrent diseases, pregnancy/delivery for in-hospital death cases

Analysis set: all patients		
Classification	*N* = 338	
Disease name	*n*	(%)
In-hospital death	63	(18.6)
Pregnancy/delivery in-hospital death^a^	0	(0.0)
Malignancy in-hospital death^b^	19	(30.2)
Malignant lymphoma	5	(7.9)
Stomach cancer	3	(4.8)
Colon cancer	3	(4.8)
Prostate cancer	3	(4.8)
Colorectal cancer	2	(3.2)
Cholangiocarcinoma	2	(3.2)
Rectal cancer	2	(3.2)
Autoimmune disease in-hospital death^b^	8	(12.7)
Rheumatoid arthritis	2	(3.2)
Primary biliary cirrhosis	2	(3.2)
Systemic lupus erythematosus	2	(3.2)
Other in-hospital death^b^	21	(33.3)
Diabetes mellitus	14	(22.2)
Bronchial asthma	5	(7.9)
Nephrotic syndrome	4	(6.3)
Hepatitis	4	(6.3)

Note: Disease names were sorted by each classification in descending order of the patient numbers and only the most frequent diseases were shown

The most frequently recorded categories of concurrent diseases were shown, and the disease names recorded in more than 3% of in-hospital deaths in these categories were shown in this table

^a^Percentage of total female patients in-hospital deaths (*N* = 26)

^b^Percentage of total in-hospital deaths (*N* = 63)

The proportion of deaths during hospitalization was 18.6% (63 patients), and of those that died, 30.2% (19 patients) had malignancy, 12.7% (8 patients) had autoimmune diseases, and 33.3% (21 patients) had other concurrent diseases at diagnosis (Table 6). Among fatal cases, only 1 patient exhibited dermatological disease in the hospitalized period.

### Thromboembolism (Table S2)

Among hospitalized patients (*N* = 328; patients who could be measured in person-year analysis, and had available admission/discharge information), acute coronary syndromes was the most frequent type (0.9%, 3 patients) among thromboembolism, with an incidence rate [100 person-years] of 8.49 (Table S2).

Among patients with thromboembolism (4.4%, 15 patients), the mean age (S.D.) was 74.9 (9.8) years, 80.0% were male and 20.0% were female, and 20.0% (3 patients) had autoimmune disease, 13.3% (2 patients) had malignancy, and 53.3% (8 patients) had other concurrent diseases. Percentages of hemostatic agent use in the patients with thromboembolism were 53.3% for tranexamic acid (8 patients) and 40.0% for bypassing agent (6 patients). Anti-thromboembolic drug use at baseline was 53.3% (8 patients) of the patients with thromboembolism.

### Bleeding (Table S3)

In patients with available data from the first admission to final discharge (328 patients), the most frequent type of bleeding was gastrointestinal bleeding with 10.1% (33 patients), followed by intramuscular bleeding with 9.1% (30 patients), and subcutaneous hemorrhage with 5.5% (18 patients). The incidence rate [person-years] was 76.43 for gastrointestinal bleeding, 72.19 for intramuscular bleeding and 38.22 for subcutaneous hemorrhage. A small number of patients reported serious bleedings such as intracranial hemorrhage (2.4%, 8 patients), retroperitoneal hemorrhage (2.1%, 7 patients), intraperitoneal hemorrhage (0.9%, 3 patients), and intrathoracic hemorrhage (0.3%, 1 patient) (Table S3).

### Infection (Table S4)

Regarding infections during the hospitalization, 12.5% (41 patients) showed bacterial infections, 5.5% (18 patients) showed septicemia, and 56.7% (186 patients) had others. The incidence rates [person-years] of infection by type were 94.83 for bacterial infections, 38.22 for septicemia, and 638.36 for others. Pneumonia was recorded in 101 patients during the hospitalization period (Table S4).

### Discussion

### Findings of this study

The observational study was conducted using a health administrative database and had some limitations like findings only from claims information. Therefore, the criteria of inclusion and exclusion were to be set stringently to exclude patients who were unlikely AHA patients. Another notable limitation was the difficulty gathering comprehensive treatment details on individual patient.

So far, no large-scale epidemiological survey on AHA has been conducted in Japan. In this study, the epidemiology and treatment patterns of the disease were investigated using accumulated cases from claims data. MDV data was collected from acute-care hospitals and suitable for understanding the actual status of AHA. After applying inclusion and exclusion criteria, 338 patients were included and analyzed as a population with AHA, and their characteristics and treatment patterns in Japanese clinical practices were investigated. Moreover, since MDV data covers 24% of all acute-care hospitals [21], nearing one-quarter of the hospitals in Japan, the annual incidence of AHA is projected be approximately 0.8 to 1.2 per million people based on the number of 338 patients enrolled in the study over a 12-year period. This incidence, mentioned in the introduction, is comparable with those of European epidemiological surveys.

### Discussion of the results

#### Patient characteristics

In previous Japanese reports, the male to female ratios were 1:0.7–0.9 [1, 9, 12], and the onset ages of AHA ranged from 12 to 92 years. From an epidemiological point of view, there were slightly more male patients, and the mean age was slightly higher in this study compared to the demographics in the previous reports. The possible reason for this may be regional variation, distinct data source or the fact that the Japanese national health insurance system and hemophilia treatment guidelines do not require patients to visit hemophilia-specialized treatment centers.

Even though, incidence of hematological malignancy such as malignant lymphoma (3.7%), leukemia (1.5%) and multiple myeloma (0.8%) was not so high compared with other solid tumors such as colorectal (15.5%), stomach (12.9%) and lung (12.5%) in Japan [26], the result from this study shows high proportion of malignant lymphoma as concurrent diseases. The proportion of hematological malignancy was relatively higher compared with solid tumors for concurrent diseases. This higher diagnosis rate of hematological malignancy may be because AHA and hematologic malignancies are treated in the same hematology department.

#### IST and hemostatic treatment

The Use of immunosuppressive agents and hemostatic agents was consistent with the previous reports [1, 4, 13]. The unique results in Japan were the use of FVIIa/FX as hemostatic agents and the predominance of PSL monotherapy as IST. The duration of oral drug administrations was 3 to 5 months. Such long-term administrations of steroids may cause many complications and result in lower ADL scores of elderly patients. Even median duration of bypassing agent administration was as short as 2.5 to 6 days, the duration of hospital stay was relative longer with a median of 62 days. This may be due to older age of AHA patients as described previously, and the underlying cause remain to be clarified in the future.

#### Rehabilitation and ADL

Rehabilitation was implemented for most of the AHA patients, and it usually took about 2 to 3 weeks to start rehabilitation from admission. Low ADL scores were seen both at the time of admission and discharge. The large number of elderly people may lead to the low ADL scores. The reason was not clear in this study that cerebrovascular rehabilitation was recorded as many as in 49 patients while intracranial bleeding was recorded only in 8 patients. The claims code for the cerebrovascular rehabilitation is not necessarily limited to cerebrovascular disease and may include neurological diseases such as multiple sclerosis.

#### Bleeding and thrombotic events

Common types of bleeding were gastrointestinal bleeding with 10.1% (33 patients), intramuscular bleeding with 9.1% (30 patients) and subcutaneous bleeding with 5.5% (18 patients). Minor symptoms such as epistaxis, hematuria and mild subcutaneous bleeding were less likely to be reported compared with severe symptoms such as intracranial hemorrhage and gastrointestinal bleeding. One possible cause was that these minor bleedings may not be recorded in the claims data (only ‘acquired hemophilia A’ may be recorded) in the medical field. Additionally, gastric ulcers in some cases may have been recorded as a disease and considered gastrointestinal bleeding for the purpose of prophylactic prescription. Furthermore, gastrointestinal bleedings may have been recorded as claims in order to prescribe medicines such as Proton Pump Inhibitor.

#### Limitation and countermeasures

Since this study relied on secondary use of a health administrative database, the following selection biases and limitations were considered.

Three selection biases need to be considered regarding the administrative data used in this study. The first was the medical institutions in the database, and the second was the study population selected from the inclusion and exclusion criteria. In order to exclude the patients who possibly were not AHA, inclusion and exclusion criteria were strictly set. However, the criteria might be so strict that it might exclude some patients with AHA. The third was the number of evaluable patients with the valid data analyzed in the several analyses, such as ADL or admission/discharge related results. Validation study is useful for assessing these biases, but MDV data is anonymized and difficult to trace back to the original data. To maximize the validity of definitions in this study, definitions validated in the previous report [27] and constructed in consultation with clinicians were used.

In addition to the biases mentioned above, there are several potential limitations to this study. First, MDV data does not include deaths occurring outside of hospitalization. In addition, patients transferred to other hospital or receiving AHA treatments in other hospital cannot be traced in the database even in the follow-up period. Deaths were not tracked outside the hospitalization, and causal relationship was not included in claims database. Also this database did not include the clinical off-label uses of treatment and investigative treatments in clinical trials, which were outside of the insurance claims coverages.

Secondly, in claims data, laboratory values such as the titer of autoantibodies against FVIII and coagulation activity are often limited or absent, so it is not possible to accurately evaluate recurrence or therapeutic effects. Therefore, those values were not evaluated in this study.

Finally, as a limitation of research design, concurrent disease information could not be obtained for patients diagnosed or treated outside the baseline period and may not be as accurate as the well accepted definitions of concurrent diseases because the disease names were recorded for insurance claims. The information was mainly from acute care hospitals in MDV data and based on the insurance claims from each hospital. It is undeniable that there are missed comorbidities. In this study, none of the patients had their onset triggered by pregnancy or delivery.

## Conclusions

Despite limitations to real-world dataset, this study provides insights into the clinical treatment of AHA in a large population over a relatively long period of time and holds great impact on the AHA clinical practices in Japan. Especially for data of rehabilitation and ADL, which are directly linked to the treatment needs for AHA, it promoted better understanding of real-world practices and fostered better solutions.

## Supplementary Information

Below is the link to the electronic supplementary material.

Supplementary file1 (DOCX 59 KB)
