# Supplementary material for: ORIHIME study: real-world treatment patterns and clinical outcomes of 338 patients with acquired hemophilia A from a Japanese administrative database
Source: Int J Hematol. 2022 Nov 4;117(1):44–55. doi: 10.1007/s12185-022-03467-w (PMC9813148; doi:10.1007/s12185-022-03467-w)
Supplement: Supplementary file 1 — Supplementary file1 (DOCX 59 KB) [file 12185_2022_3467_MOESM1_ESM.docx]

Supporting Information

Table S1: ADL Scores at first admission and final discharge

Analysis set: All evaluable patients (patients with non-missing data for all 10 items)

|  |  |  |  |  |  |  |
| --- | --- | --- | --- | --- | --- | --- |
|  |  |  | N=196 | | | |
|  |  |  | At first admission | | At final discharge | |
|  |  |  | n | (%) | n | (%) |
|  |  |  |  |  |  |  |
| Feeding |  |  |  |  |  |  |
|  | 10 | Independence | 122 | (62.2) | 133 | (67.9) |
|  | 5 | Partial assistance | 33 | (16.8) | 26 | (13.3) |
|  | 0 | Total assistance | 41 | (20.9) | 37 | (18.9) |
| Transfer |  |  |  |  |  |  |
|  | 15 | Independence | 86 | (43.9) | 112 | (57.1) |
|  | 10 | Mild partial assistance | 49 | (25.0) | 31 | (15.8) |
|  | 5 | Severe partial assistance | 10 | (5.1) | 7 | (3.6) |
|  | 0 | Total assistance | 51 | (26.0) | 46 | (23.5) |
| Grooming |  |  |  |  |  |  |
|  | 5 | Independence | 106 | (54.1) | 118 | (60.2) |
|  | 0 | Partially assisted/fully assisted | 90 | (45.9) | 78 | (39.8) |
| Toilet use |  |  |  |  |  |  |
|  | 10 | Independence | 98 | (50.0) | 116 | (59.2) |
|  | 5 | Partial assistance | 42 | (21.4) | 29 | (14.8) |
|  | 0 | Total assistance | 56 | (28.6) | 51 | (26.0) |
| Bathing |  |  |  |  |  |  |
|  | 5 | Independence | 89 | (45.4) | 102 | (52.0) |
|  | 0 | Partially assisted/fully assisted | 107 | (54.6) | 94 | (48.0) |
| Mobility |  |  |  |  |  |  |
|  | 15 | Independence | 88 | (44.9) | 108 | (55.1) |
|  | 10 | Walk alone with assistance | 31 | (15.8) | 27 | (13.8) |
|  | 5 | Independence in wheelchairs | 13 | (6.6) | 7 | (3.6) |
|  | 0 | Total assistance | 64 | (32.7) | 54 | (27.6) |
| Stairs |  |  |  |  |  |  |
|  | 10 | Independence | 87 | (44.4) | 102 | (52.0) |
|  | 5 | Partial assistance | 28 | (14.3) | 27 | (13.8) |
|  | 0 | Total assistance | 81 | (41.3) | 67 | (34.2) |
| Dressing |  |  |  |  |  |  |
|  | 10 | Independence | 99 | (50.5) | 114 | (58.2) |
|  | 5 | Partial assistance | 41 | (20.9) | 30 | (15.3) |
|  | 0 | Total assistance | 56 | (28.6) | 52 | (26.5) |
| Bowels | | |  |  |  |  |
|  | 10 | Independence | 123 | (62.8) | 126 | (64.3) |
|  | 5 | Partial assistance | 20 | (10.2) | 18 | (9.2) |
|  | 0 | Total assistance | 53 | (27.0) | 52 | (26.5) |
| Bladder | |  |  |  |  |  |
|  | 10 | Independence | 119 | (60.7) | 127 | (64.8) |
|  | 5 | Partial assistance | 22 | (11.2) | 19 | (9.7) |
|  | 0 | Total assistance | 55 | (28.1) | 50 | (25.5) |
|  |  |  |  |  |  |  |
| Total score |  |  |  |  |  |  |
|  |  | Less than 70 points | 93 | (47.4) | 76 | (38.8) |
|  |  | 70 points | 3 | (1.5) | 3 | (1.5) |
|  |  | 75 points | 4 | (2.0) | 6 | (3.1) |
|  |  | 80 points | 8 | (4.1) | 3 | (1.5) |
|  |  | 85 points | 3 | (1.5) | 3 | (1.5) |
|  |  | 90 points | 3 | (1.5) | 4 | (2.0) |
|  |  | 95 points | 5 | (2.6) | 4 | (2.0) |
|  |  | 100 points | 77 | (39.3) | 97 | (49.5) |
|  |  |  |  |  |  |  |
|  |  |  |  | |  | |
|  |  | Min | 0 | | 0 | |
|  |  | Q1 | 15.0 | | 25.0 | |
|  |  | Median | 75.0 | | 95.0 | |
|  |  | Q3 | 100.0 | | 100.0 | |
|  |  | Max | 100 | | 100 | |
|  |  |  |  |  |  |  |

Table S2: Thromboembolism during hospitalization

Analysis set: All evaluable patients (patients with data at first admission and at final discharge of the hospital)

|  |  |  |  |  |  |  |
| --- | --- | --- | --- | --- | --- | --- |
|  |  |  |  |  |  |  |
| Type of thromboembolism | |  | N=328 | | | During hospitalization |
|  |  |  |  |  |  | Incidence |
|  |  |  | n | (%) | Number  of events | [incidence/100 person-year] |
|  |  |  |  |  |  |  |
| The sum of the periods for each patient [person-year] | | | | | | 70.65 |
|  |  |  |  |  |  |  |
| Acute coronary syndromes | |  | 3 | (0.9) | 6 | 8.49 |
| Pulmonary embolism | |  | 1 | (0.3) | 1 | 1.42 |
| Other | |  | 1 | (0.3) | 1 | 1.42 |
|  |  |  |  |  |  |  |

Note: Of the analysis population (N=338), 10 patients had no summary data at hospital discharge.

The patients number of this table is N=328.

A thromboembolism was counted as 1 event if the use of an antithrombotic agent was confirmed in the same month as the record of the thromboembolism on the patient during hospitalization. The first use of an antithrombotic agent was counted as 1 event, and the agent use within 1 month of the first use was considered as the same thromboembolism. Any antithrombotic agents used 1 month or later after the first use was counted as a new thromboembolism. Subsequent events were counted in the same way during the follow-up period.

Table S3: Bleeding during hospitalization

Analysis set: All evaluable patients (patients with data at first admission and at final discharge of the hospital)

|  |  |  |  |  |  |
| --- | --- | --- | --- | --- | --- |
|  |  |  |  |  |  |
| Site of bleeding | | N=328 | | | During hospitalization |
|  |  |  |  |  | Incidence |
|  |  | n | (%) | Number  of events | [incidence/100 person-year] |
|  |  |  |  |  |  |
| The sum of the periods for each patient [person-year] | |  |  |  | 70.65 |
|  |  |  |  |  |  |
| Gastrointestinal bleeding | | 33 | (10.1) | 54 | 76.43 |
| Intramuscular bleeding | | 30 | (9.1) | 51 | 72.19 |
| Subcutaneous hemorrhage | | 18 | (5.5) | 27 | 38.22 |
| Intracranial hemorrhage | | 8 | (2.4) | 11 | 15.57 |
| Retroperitoneal bleeding | | 7 | (2.1) | 7 | 9.91 |
| Haematuria | | 5 | (1.5) | 5 | 7.08 |
| Intraperitoneal hemorrhage | | 3 | (0.9) | 4 | 5.66 |
| Genital bleeding | | 2 | (0.6) | 2 | 2.83 |
| Intrathoracic hemorrhage | | 1 | (0.3) | 1 | 1.42 |
| Pharyngeal hemorrhage | | 1 | (0.3) | 1 | 1.42 |
| Intra-articular hemorrhage | | 1 | (0.3) | 1 | 1.42 |
| Epistaxis | | 1 | (0.3) | 1 | 1.42 |
| Airway bleeding and pulmonary hemorrhage | | 0 | (0.0) | 0 | 0.00 |
| Haemoptysis | | 0 | (0.0) | 0 | 0.00 |
|  |  |  |  |  |  |

Note: Of the analysis population (N=338), 10 patients had no summary data at hospital discharge.

The patients number of this table is N=328.

A bleeding was counted as 1 event if the use of a hemostatic agent was confirmed in the same month as the record of the bleeding on the patient during hospitalization. The first use of a hemostatic agent was counted as 1 event and a use of the agent within 1 month of the first use was considered as the same bleeding. Any hemostatic agent used 1 month or later after the first use was counted as a new bleeding. Subsequent events were counted in the same way during the follow-up period.

Table S4: Infection during hospitalization

Analysis set: All evaluable patients (patients with data at first admission and at final discharge of the hospital)

|  |  |  |  |  |  |  |
| --- | --- | --- | --- | --- | --- | --- |
|  |  |  |  |  |  |  |
| Type of infection | |  | N=328 | |  | During hospitalization |
|  |  |  |  |  |  | Incidence |
|  |  |  | n | (%) | Number  of events | [incidence/100 person-year] |
|  |  |  |  |  |  |  |
| The sum of the periods for each patient | | |  |  | | 70.65 |
| [person-year] | | |  |  | |  |
|  |  |  |  |  |  |  |
| Bacterial infections | |  | 41 | (12.5) | 67 | 94.83 |
| Septicemia | |  | 18 | (5.5) | 27 | 38.22 |
| Other | |  | 186 | (56.7) | 451 | 638.36 |
|  |  |  |  |  |  |  |

Note: Of the analysis population (N=338), 10 patients had no summary data at hospital discharge.

The patients number of this table is N=328.

The number of patients with pneumonia during the follow-up period was 146, and the number of patients with pneumonia during hospitalization was 101.

An infection was counted as 1 event if the use of an infectious treatment agent was confirmed in the same month as the record of the infection on the patient during hospitalization. The first use of an infectious treatment agent was counted as 1 event and the agent use within 1 month of the first use was considered as the same infection. Any infectious therapeutic agent used 1 month or later after the first use was counted as a new infection. Subsequent events were counted in the same way during the follow-up period.
